# Clinical Survey of Decreased Blood Flow Rate in Continuous Renal Replacement Therapy: A Retrospective Observational Study

**DOI:** 10.1155/2019/2842313

**Published:** 2019-11-20

**Authors:** Makoto Harada, Masafumi Ooki, Kaede Kohashi, Tohru Ichikawa, Mamoru Kobayashi

**Affiliations:** ^1^Department of Nephrology, Nagano Red Cross Hospital, 5-22-1, Wakasato, Nagano 380-8582, Japan; ^2^Department of Nephrology, Shinshu University School of Medicine, 3-1-1, Asahi, Matsumoto 390-8621, Japan

## Abstract

**Background:**

Continuous renal replacement therapy (CRRT) is an essential procedure for patients with acute kidney injury in intensive care. It is important to maintain an adequate blood flow rate during CRRT. Several previous studies have reported the relationships between blood flow rate and filter lifespan, or circuit life, in CRRT. Here, we aim at elucidating the incidence and factors associated with a decreased blood flow rate in CRRT.

**Methods:**

This is a retrospective observational study. From January 2014 to June 2017, 119 patients who underwent CRRT in the intensive care unit were enrolled. The definition of a decreased blood flow rate included situations in which the medical staff needed to decrease the blood flow volume. We statistically analyzed the association of the decreased blood flow rate with patients' clinical characteristics.

**Results:**

Of 119 patients, 52 required a decreased blood flow rate during CRRT. Almost half of the cases occurred within one day of starting CRRT. None of the clinical factors (age, sex, height, sequential organ failure assessment (SOFA) score, catheter position, systemic infection, albumin, hemoglobin, and activating coagulation time) were significantly associated with decreased blood flow rate.

**Conclusions:**

A decreased blood flow rate often occurs during CRRT. Clinical factors significantly associated with the occurrence of the decreased blood flow rate were not detected in the current study. Further investigation regarding the occurrence of a decreased blood flow is warranted.

## 1. Introduction

In intensive care units, the frequency of acute kidney injury is approximately 30–40% [1], and continuous renal replacement therapy (CRRT) is often required [2]. A previous study revealed that in patients who developed acute renal failure, 72.4% required renal replacement therapy. In addition, among those, almost 80% of patients required CRRT [2]. Performing CRRT requires a vascular access catheter insertion into a central vein. Regarding the position of catheter insertion, the right internal jugular or femoral vein is typically recommended [3]. As for catheter-related complications in CRRT, catheter-related bloodstream infections are well known and often researched [4–6]. A decreased blood flow rate is another important problem that can occur during CRRT. Decreased blood flow rate from the vascular access catheter includes the several complications, such as decreased CRRT efficiency, increase of therapy downtime, potential loss of solute, acid-base and fluid balance control, increased therapy cost, and a reduction in circuit or filter life, resulting in patients' blood loss. Several studies have investigated the importance of blood flow rate in CRRT [7–11]. Baldwin et al. investigated the relationship between the decrease in the blood flow rate and circuit life in CRRT [8]. They demonstrated that an undetected reduction in the blood flow rate occurs and that set blood flow is inaccurate [8]. In addition, frequent reductions in blood flow rates were significantly associated with a decreased circuit life [8]. As several studies indicated that a higher blood flow rate may be associated with the improvement in circuit life in CRRT [9, 10], a randomized control trial was performed to compare the two types of blood flow rate in CRRT [7]. However, although it is believed that the circuit life in the higher blood flow rate (250 ml/min) group is longer than that in the lower blood flow rate (150 ml/min) group, there was no significant difference between the groups [7]. With respect to the blood flow rate in CRRT, most studies have discussed the relationship between the blood flow rate and circuit life. However, studies investigating the factors underlying the blood flow rate are rarely conducted. In this study, we discuss the relationship between the blood flow rate itself and the associated factors. Here, we aimed at clarifying the frequency of decreased blood flow rates from the hemodialysis catheter in CRRT and its associated clinical factors. In relation to clinical factors, we investigated factors associated with patients' characteristics and factors associated with the clinicians who inserted the vascular access catheter.

## 2. Materials and Methods

### 2.1. Study Design

This is a retrospective observational study that occurred in a single center in Japan. Patients underwent CRRT in the intensive care unit of Nagano Red Cross Hospital from January 2014 to June 2017. The exclusion criteria consisted of patients whose clinical data were not fully obtained or patients younger than 20 years old. The study protocol was approved by the institutional review board of the ethical committee at Nagano Red Cross Hospital and was conducted in accordance with the principles of the Declaration of Helsinki, as revised in 2013. The patients' data were obtained from medical records.

### 2.2. Confirmation of Catheter Position

After insertion of the vascular access catheter, the position of the catheter tip was checked by X-ray, and we confirmed that the position was appropriate in all cases. The catheter tip was placed at the superior vena cava when the vascular access catheter was inserted in the left or right jugular vein, at the inferior vena cava when it was inserted in the right femoral vein, or at the inferior vena cava or left common iliac vein when it was inserted in the left femoral vein.

### 2.3. Setup of CRRT

All patients were treated with continuous venovenous hemodialysis (CVVHD) or continuous venovenous hemodiafiltration (CVVHDF). All CVVHDF procedures were performed using the postdilution protocol. For the setup of CRRT, the blood flow rate at the start of CRRT and the type of anticoagulant were uniform in all patients. The blood flow rate at the start time of CRRT was 100 mL/min, and nafamostat mesylate was used as an anticoagulant in all patients. The amount of the nafamostat mesylate was determined by the clinical physician according to the patient's medical chart and activated coagulation time. Nafamostat mesylate was administered from the CRRT machine before blood entered the hemofilter. The vascular access catheter used in the current study was also uniform for all patients. The clinical physician who inserted the vascular access catheter decided whether to double or triple the lumen catheter. The urokinase-coated temporary vascular access catheter (Blood Access UK-Catheter kit, NIPRO, Tokyo, Japan) was used universally in all patients. The size (gauge) of the catheters was 11.5 Fr. The CRRT machines used in this study were the JUN-505 (Junken Medical, Tokyo, Japan), JUN55X (Junken Medical, Tokyo, Japan), or AcuFil Multi 55X-II (Japan Lifeline, Tokyo, Japan). These machines perform CRRT in a similar manner and require the same blood circuits.

### 2.4. Definitions

A decreased blood flow rate from the vascular access catheter was defined as an adequate blood flow rate that could not be continuously sustained (blood flow rate at the start of CRRT was 100 mL/min in all cases, and a decrease in the blood flow rate would be a 10% reduction in the blood flow rate for 10 minutes) and/or required intervention by medical staff (changing the connection of the catheter or inserting a new vascular access catheter). We collected data on the decrease in the blood flow rate according to the following situations: the alarm raised due to inadequate blood flow rate continues and the rate does not improve for at least 10 minutes despite the administration of relevant interventions. The decrease in the blood flow rate due to the elevation of transmembrane pressure (TMP) and the arterial or venous side pressure was not considered. These decisions (10% reduction of blood flow rate) were made by medical or nursing staff. Catheter-related bloodstream infections were defined when clinicians diagnosed infections caused by the vascular access catheter whether or not bacteria were detected in blood cultures.

### 2.5. Statistical Analysis

Continuous variables are presented as the median and interquartile range. Categorical variables are presented as number (*n*) and frequency (%). Clinical data between two groups of patients were compared, with group 1 being patients' with a decreased blood flow rate in the therapeutic course of CRRT and group 2 being patients' with a preserved blood flow rate. Continuous variables between both groups were compared using the Mann–Whitney *U* test, and categorical variables were compared using the Fisher exact probability test. Factors considered to be associated with the occurrence of decreased blood flow rate from the vascular access catheter were investigated by performing Cox hazard regression analyses. We performed multivariate analyses of six models composed of the following factors: age, gender, height, sequential organ failure assessment (SOFA) score, serum albumin level, hemoglobin, activated coagulation time, sepsis or severe infectious diseases, heart failure, CVVHDF, and catheter position. In addition, we evaluated the clinicians who inserted the vascular access catheter. A *p* value of <0.05 was considered to indicate statistical significance. Analyses were performed using EZR (Saitama Medical Center, Jichi Medical University, Saitama, Japan), a graphical user interface for *R* (The *R* Foundation for Statistical Computing, Vienna, Austria) [12].

## 3. Results

The current study included 119 patients. Patients' characteristics are presented in Table 1. The median age of the patients was 71 years, and 69.7% of the patients were male. The median SOFA score was 12. Diagnosis and indication of CRRT are presented in Tables 1 and 2. Sepsis or severe infectious disease was the main reason for starting CRRT, followed by heart failure. With respect to the vascular access catheter, 73.1% of patients required a triple lumen catheter. In almost 80% of the patients, the vascular access catheter was inserted in the femoral vein (70 patients in the right and 25 in the left femoral vein). The catheter lengths inserted in the jugular vein were 15 cm (20 cases), 14 cm (3 cases), and 12 cm (1 case), while the catheter lengths inserted in the femoral vein were 30 cm (2 cases), 25 cm (74 cases), 24 cm (10 cases), 23 cm (2 cases), 22 cm (1 case), 20 cm (3 cases), 18 cm (1 case), and 15 cm (2 cases). Of the 119 patients, 100 required CHDF, while 19 patients required CVVHD. CVVHDF was performed by the postdilution method in all cases (Tables 1 and 2). The primary dose of quantity of filtration (Qf), quantity of dialysis fluid (Qd), and quantity of ultrafiltration (Quf) for CVVHDF and CVVHD was recorded. In patients who required CVVHDF, the mean initial Qf dose was 206 ml/h, Qd dose was 492 ml/h, and Quf dose was 12 ml/h, while in CVVHD, the mean initial Qd dose was 621 ml/h and Quf dose was 28 ml/h. A decreased blood flow rate was noted in 52 (43.7%) patients.

Almost half of the patients died during hospitalization. Age, height, body weight, BMI, SOFA score, hemoglobin, albumin, C-reactive protein levels, and activated coagulation time were not significantly different regardless of whether the patients developed a decrease in the blood flow rate (Table 2). In addition, male gender, diabetes mellitus, intubation, diagnosis at the start of CRRT, type of hemofilter, mode of CVVHDF, use of a triple lumen catheter, position of catheter insertion, and all-cause death in CRRT and hospitalization were not significantly different between the two groups (Table 2). Factors associated with the clinicians who inserted the vascular access catheter were also not different between the groups (Table 2). Systolic and diastolic blood pressure, heart rate, and frequency of catheter-related bloodstream infection were higher in patients who developed a decreased blood flow rate (group 1) than those who (group 2) did not (Table 2).

The univariate Cox hazard regression analysis indicated that there was no significant association between the development of a decreased blood flow rate from the vascular access catheter and catheter position, age, gender, height, SOFA score, sepsis or severe infectious diseases, heart failure, mode of CRRT (CVVHDF), serum albumin level, hemoglobin level, or activated coagulation time (Table 3). In addition, the characteristics of clinicians who inserted the vascular access catheter were also not significantly associated with the development of a decreased blood flow rate from the vascular access catheter (Table 3).

Multivariate Cox regression analyses demonstrated that there were no significant associations between the occurrence of a decreased blood flow rate and the clinical characteristics of the patient (serum albumin level, hemoglobin, activated coagulation time, sepsis or severe infectious diseases, heart failure, CVVHDF, and catheter position) when adjusted for general confounding factors, such as age, gender, height, and SOFA score (Table 4).

**Figure 1:**
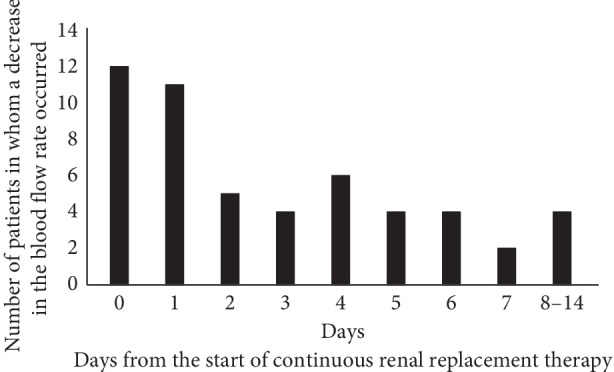
Number of days between initial catheter insertion and the occurrence of a decreased blood flow rate during continuous renal replacement therapy (CRRT).

Duration between catheter insertion and the occurrence of a decreased blood flow rate data is presented in Figure 1. Almost half of the cases developed a decreased blood flow rate within one day from the start of CRRT.

## 4. Discussion

The incidence of a decreased blood flow rate was high, at 43.7% in patients who required CRRT, and in most cases developed within one day after the start of CRRT. There were no significant associations between the development of a decreased blood flow from the vascular access catheter and general clinical factors, including catheter position.

In regard to catheter-related problems, catheter-related bloodstream infections, complications accompanying the insertion of the central venous catheter, and venous thrombosis have been previously reported [4, 5]. Recent studies indicated that obesity is one of the risk factors for developing catheter-related bloodstream infections [3, 5]. The position of the vascular access catheter, whether placed in the internal jugular or femoral vein, was not significantly associated with the incidence of infections [4–6]. However, although the occurrence of decreased blood flow from the vascular access catheter is important in terms of complications related to the vascular access catheter, little knowledge exists on this topic. The occurrence of the decreased blood flow rate from the vascular access catheter may be a serious problem for patients who require CRRT. CRRT can be less efficient than expected due to interruptions such as the diagnostic investigations, clotting, changing circuits, and surgery [13]. In addition, when a decreased blood flow rate occurs, CRRT efficiency is further reduced. Elucidation of the causes of the decrease in the blood flow rate or a method for predicting the occurrence of the decrease in the blood flow rate in CRRT is eagerly awaited in clinical settings of intensive care. With respect to the blood flow rate in CRRT, most studies have discussed the relationship between blood flow rate and circuit life. However, research that investigated factors associated with the occurrence of blood flow rate is rarely conducted. In this study, we discussed the relationship between blood flow rates and relating factors. In factors considered to be associated with the occurrence of decrease in blood flow, height may be of consideration, as shorter patients can experience a more deeply inserted vascular access catheter, which would be favorable for CRRT. However, our results indicated that it was not significantly associated with the occurrence of a decreased blood flow. In regard to coagulation in the catheter lumen, although we analyzed the coagulation ability by evaluating the activated coagulation time, we did not find a significant association (the range of dose for nafamostat mesylate was 0 to 40 mg/h, and it was determined according to the activated coagulation time). SOFA score, serum albumin level, hemoglobin level, and diagnosis at the time of starting CRRT (such as sepsis or severe infectious diseases, heart failure, and after cardiac surgery) were also analyzed. It is possible that low serum albumin level, anemia, and sepsis or severe infectious disease may be related to the development of hypovolemic outcomes resulting in decreased blood flow. However, these factors were not significantly associated with the outcome of this study. Although systolic and diastolic blood pressure and heart rate were significantly different between patients who developed a decreased blood flow rate and those who did not, these factors are strongly influenced by the use and dosage of catecholamine. The SOFA score includes the use and dosage of catecholamine, and we did not detect a significant difference in SOFA scores between both groups. Therefore, we believe that disease severity, including blood pressure and heart rate, was not substantially associated with a decreased blood flow rate. There are several studies that have evaluated the training of clinicians for catheter insertion [14–16]. Thus, the skills of catheter insertion may be associated with the occurrence of decreased blood flow from the vascular access catheter. We analyzed factors related to clinicians who inserted the vascular access catheter by monitoring their overall experience and their medical specialties. However, neither were significantly associated with a decreased blood flow rate. In addition, although the vascular access catheters used in the current study were uniform for all patients, in recent years, there has been an increase in the number of vascular access catheter types, which differ with size (external or internal diameter) or structure (lumen shape or design of holes) [17, 18]. It is possible that differences in the type of vascular access catheter may be associated with the decreased blood flow rate.

Inadequate blood flow rate or decrease in the blood flow rate in CRRT is a noticeable and serious problem. Its incidence may be higher than that considered by us. The reason for the inadequate blood flow rate or decrease in the blood flow rate may be that most medical centers, except for those in Japan, adopted a higher blood flow rate in CRRT. The patients who receive CRRT where the blood flow rate is low are less likely to develop an inadequate flow rate than those who receive CRRT where the blood flow rate is high. In our study, a decrease in the blood flow rate was noted in 43.7% of the patients; therefore, an inadequate blood flow rate is more likely to develop frequently in patients in foreign medical centers than those in centers in Japan.

In the clinical setting, a decrease in the blood flow rate in CRRT is an undesirable situation because this may shorten the filter or circuit life and decrease CRRT efficiency and ultrafiltration. Therefore, once the blood flow rate decreases, the vascular access catheter should be ideally reinserted or the central vein in which the vascular access catheter was inserted should be changed. However, for patients with severe physiological conditions often complicated with remarkable thrombocytopenia or coagulation disorder, the insertion of the vascular access catheter is accompanied by the risk of bleeding. Thus, in some cases, the decrease in the blood flow rate may be allowed in clinical settings.

This study had several limitations. It was a retrospective observational study, with a small sample size. Although we investigated the clinical factors that were associated with decreased blood flow volume from the vascular access catheter, we could not investigate the position of the tip of the catheter in the central vein. There are several studies that have discussed the optimal positioning of the central venous catheter tip [19, 20]. Although the position of the catheter tip was generally appropriate, the intravenous positioning, such as in venous valves, venous branches, or pressure onto the venous wall from outside organs, could be associated with the development of a decreased blood flow. The type of catheter used was uniform across this study. Because there are many types of vascular access catheters, the incidence of a decreased blood flow rate may be different depending on the selected type and this variable may need further investigation, in future studies.

The size of the inferior vena cava and central venous pressure is an important marker for evaluating the volume status. However, we did not obtain complete data for these parameters. The marker of volume status may be the predictive marker of the occurrence of a decreased blood flow rate. Prospective studies must be conducted to assess this. There was no unified protocol for the evaluation of catheter function (e.g., a unified absorption test for vascular access catheter). In the present study, identification of catheter function at the time of catheter insertion depended on the physician who inserted the catheter. This may be a limitation of our study.

We attempted to obtain the data on the circuit or filter lives of these circuits. However, we could not obtain the complete data. In addition, CRRT was interrupted in some patients because of examinations (e.g., computed tomography, magnetic resonance imaging, angiography, and other such examinations) or therapeutic interventions (e.g., operation, catheter intervention, and other such interventions). Therefore, it was difficult to evaluate the substantial filter life in CRRT. This was also a limitation of the current retrospective study.

Due to the retrospective nature of this study, we cannot clearly define the algorithm for blood flow reduction, and it was challenging to clarify the trigger for the decrease in the blood flow rate. This is a limitation of our study. However, all events (occurrence of a decrease in the blood flow rate) counted were defined as an inadequate blood flow rate from the time of insertion of the vascular access catheter. The decrease in the blood flow rate due to the elevation of transmembrane pressure (TMP) and the arterial or venous side pressure was not considered. In addition, the alarm raised due to inadequate blood flow rate continues and the rate does not improve for at least 10 minutes despite the administration of relevant interventions.

For statistical analyses, in order to investigate the factors associated with the occurrence of a decreased blood flow rate, we planned to perform Cox hazard regression analyses after adjusting for four general confounding factors (age, sex, SOFA score, and position of the inserted catheter). Finally, as a decrease in the blood flow rate was noted in 52 patients, we could perform the statistical analyses, as planned. We did not completely calculate the required sample size before the commencement of data collection because the current study was a single-center study and the patient number was originally limited. This is a statistical limitation of the study.

## 5. Conclusions

Almost half of the patients who required CRRT experienced a decreased blood flow rate, and inadequate blood flow rate or decreased blood flow rate in CRRT is a noticeable and serious problem. Although it is useful to predict the occurrence of decreased blood flow from the vascular access catheter in CRRT, factors that could help in this regard could not be uncovered in the current study. In particular, evaluation of volume status using central venous pressure or type of vascular access catheter may be one of the promising factors associated with the occurrence of a decrease in the blood flow rate. Therefore, further investigations and prospective studies on the occurrence of the decreased blood flow rate from the vascular access catheter are warranted.

## Figures and Tables

**Table 1:** Clinical characteristics of the 119 enrolled patients.

Clinical characteristics		
Age (years)	71	60–78
Male (*n*, %)	83	69.7
Height (cm)	163.0	155.5–170.0
Body weight (kg)	60.0	51.4–70.0
BMI (kg/m^2^)	22.6	20.0–26.0
Diabetes mellitus (*n*, %)	41	34.4
SBP (mmHg)	98	83–119
DBP (mmHg)	58	46–64
HR (/min)	93	80–113
Intubation (*n*, %)	42	35.3
SOFA score	12	10–15
Diagnosis at the time of starting CRRT		
Sepsis or severe infectious diseases (*n*, %)	39	32.8
Heart failure (*n*, %)	34	28.6
After cardiovascular surgery (*n*, %)	25	21.0
Hepatic failure (*n*, %)	2	1.7
Mainly renal failure (*n*, %)	5	4.2
Tumor lysis syndrome (*n*, %)	2	1.7
Others (*n*, %)	12	10.1
Laboratory data		
Alb (g/dL)	2.5	2.1–3.0
Hb (g/dL)	10.6	8.8–12.5
CRP (mg/dL)	9.9	4.3–18.8
Details of catheter		
Triple lumen catheter (*n*, %)	87	73.1
Catheter position		
Femoral vein (*n*, %)	95	79.8
Jugular vein (*n*, %)	24	20.2
Condition of CRRT		
Mode of CRRT		
CVVHDF (*n*, %)	100	84.0
CVVHD (*n*, %)	19	16.0
Type of hemofilter		
PMMA (*n*, %)	51	42.9
CTA (*n*, %)	54	45.4
PS (*n*, %)	10	8.4
PES (*n*, %)	3	2.5
Others (*n*, %)	1	0.8
ACT (sec)	282	243–350
Clinical outcome		
Decrease in the blood flow rate (*n*, %)	52	43.7
Catheter-related bloodstream infection (*n*, %)	20	16.8
All-cause death during CRRT (*n*, %)	55	46.2
All-cause death during hospitalization (*n*, %)	62	52.1
Physician who inserted hemodialysis catheter		
Nephrologist (*n*, %)	66	55.5
Over 10 years of experience (*n*, %)	52	43.7

Continuous variables are presented as the median and interquartile range. Categorical variables are presented as number (*n*) and frequency (%). ACT, activating coagulation time; Alb, albumin; BMI, body mass index; CRP, C-reactive protein; CRRT, continuous renal replacement therapy; CTA, cellulose triacetate; CVVHD, continuous venovenous hemodialysis; CVVHDF, continuous venovenous hemodiafiltration; DBP, diastolic blood pressure; Hb, hemoglobin; HR, heart rate; PES, polyethersulfone; PMMA, polymethylmethacrylate; PS, polysulfone; SBP, systolic blood pressure; SOFA score, sequential organ failure assessment score.

**Table 2:** Comparison of clinical data between the two groups. Group 1: patients with a decreased blood flow rate during the therapeutic course of CRRT. Group 2: patients with a preserved blood flow rate.

Clinical characteristics	Group 1		Group 2		*p* value
	*n* = 52		*n* = 67		
Age (years)	73	57–78	69	62–77	0.62
Male (*n*, %)	37	71.2	46	68.7	0.84
Height (cm)	164.4	158.0–170.5	160.5	152.8–168.8	0.10
Body weight (kg)	62.3	52.1–69.7	58	50.2–70.0	0.56
BMI (kg/m2)	22.4	19.6–26.2	23.2	20.6–25.8	0.76
Diabetes mellitus (*n*, %)	20	38.5	21	31.3	0.44
SBP (mmHg)	106	90–125	93	79–114	0.012
DBP (mmHg)	60	50–68	52	44–62	0.034
HR (/min)	102	85–120	91	69–106	0.016
Intubation (*n*, %)	17	32.7	25	37.3	0.70
SOFA score	12	10–15	12	10–15	0.82
Diagnosis at the time of starting CRRT					
Sepsis or severe infectious diseases (*n*, %)	15	28.8	24	35.8	0.44
Heart failure (*n*, %)	14	26.9	20	29.9	0.84
After cardiovascular surgery (*n*, %)	15	28.8	10	14.9	0.07
Hepatic failure (*n*, %)	0	0	2	3.0	0.50
Mainly renal failure (*n*, %)	3	5.8	2	3.0	0.65
Tumor lysis syndrome (*n*, %)	0	0	0	0	0.50
Laboratory data					
Alb (g/dL)	2.5	2.1–2.9	2.6	2.2–3.0	0.70
Hb (g/dL)	10.7	8.9–12.4	10.3	8.5–12.6	0.57
CRP (mg/dL)	11.8	5.7–19.1	8.0	3.6–17.6	0.38
Details of catheter					
Triple lumen catheter (*n*, %)	41	78.8	46	68.7	0.30
Catheter position					
Femoral vein (*n*, %)	44	84.6	51	76.1	0.36
Jugular vein (*n*, %)	8	15.4	16	23.9	0.36
Condition of CRRT					
Mode of CRRT					
CVVHDF (*n*, %)	44	84.6	56	83.6	1.00
CVVHD (*n*, %)	8	15.4	11	16.4	1.00
Type of hemofilter					
PMMA (*n*, %)	20	38.5	31	46.3	0.46
CTA (*n*, %)	26	50.0	28	41.8	0.46
PS (*n*, %)	5	9.6	5	7.5	0.75
PES (*n*, %)	1	1.9	2	3.0	1.00
ACT (sec)	282	241–344	282	247–355	0.77
Clinical outcome					
Catheter-related bloodstream infection (*n*, %)	13	25.0	7	10.4	0.048
All-cause death during CRRT (*n*, %)	22	42.3	33	49.3	0.47
All-cause death during hospitalization (*n*, %)	26	50.0	36	53.7	0.72
Physician who inserted hemodialysis catheter					
Nephrologist (*n*, %)	30	57.7	36	53.7	0.71
Over 10 years of experience (*n*, %)	25	48.1	27	40.3	0.46

Continuous variables are presented as the median and interquartile range. Categorical variables are presented as number (*n*) and frequency (%). Continuous variables between two groups were compared using the Mann–Whitney *U* test, and categorical variables were compared using the Fisher exact probability test. ACT, activating coagulation time; Alb, albumin; BMI, body mass index; CRP, C-reactive protein; CRRT, continuous renal replacement therapy; CTA, cellulose triacetate; CVVHD, continuous venovenous hemodialysis; CVVHDF, continuous venovenous hemodiafiltration; DBP, diastolic blood pressure; Hb, hemoglobin; HR, heart rate; PES, polyethersulfone; PMMA, polymethylmethacrylate; PS, polysulfone; SBP, systolic blood pressure; SOFA score, sequential organ failure assessment score.

**Table 3:** Clinical factors associated with the development of decreased blood flow rate, evaluated by univariate Cox hazard regression analyses.

	Univariate analysis		
	HR	95% CI	*p* value
Age	1.01	0.99–1.03	0.48
Male	1.02	0.56–1.89	0.94
Height	1.01	0.99–1.04	0.30
SOFA score	1.03	0.95–1.11	0.52
Sepsis or severe infectious diseases	0.87	0.47–1.58	0.64
Heart failure	0.85	0.46–1.58	0.61
Catheter position (jugular vein)	0.64	0.30–1.36	0.24
CVVHDF	1.04	0.49–2.21	0.92
ACT	1.00	0.99–1.01	0.53
Alb	1.05	0.71–1.57	0.80
Hb	1.07	0.96–1.20	0.21
Nephrologist inserted HD catheter	0.93	0.54–1.62	0.80
Physician (over 10 years of experience) inserted HD catheter	1.24	0.71–2.14	0.44

ACT, activating coagulation time; Alb, albumin; CI, confidence interval; CVVHDF, continuous venovenous hemodiafiltration; Hb, hemoglobin; HD, hemodialysis; HR, hazard ratio; SOFA score, sequential organ failure assessment score.

**Table 4:** Clinical factors associated with the development of the decreased blood flow rate, evaluated by multivariate Cox regression analyses.

	Multivariate analysis		
	HR	95% CI	*p* value
*Model 1*			
Age	1.01	0.99–1.03	0.32
Male	1.07	0.57–2.00	0.84
SOFA score	1.04	0.96–1.13	0.33
Catheter position (jugular vein)	0.62	0.29–1.33	0.22
ACT	1.00	0.99–1.01	0.69

*Model 2*			
Age	1.02	0.99–1.04	0.07
Height	1.02	0.99–1.05	0.19
SOFA score	1.04	0.96–1.14	0.34
Catheter position (jugular vein)	0.53	0.25–1.15	0.11
Hb	1.13	0.99–1.28	0.05

*Model 3*			
Age	1.02	0.99–1.04	0.17
Height	1.02	0.99–1.05	0.22
SOFA score	1.04	0.95–1.13	0.45
Catheter position (jugular vein)	0.60	0.28–1.28	0.19
Alb	1.11	0.73–1.68	0.64

*Model 4*			
Age	1.02	0.99–1.04	0.17
Height	1.02	0.99–1.05	0.23
SOFA score	1.03	0.95–1.12	0.50
Catheter position (jugular vein)	0.59	0.28–1.27	0.18
Sepsis or severe infectious diseases	0.90	0.48–1.67	0.72

*Model 5*			
Age	1.02	0.99–1.04	0.16
Height	1.02	0.99–1.05	0.18
SOFA score	1.03	0.95–1.12	0.50
Catheter position (jugular vein)	0.61	0.28–1.30	0.20
Heart failure	0.82	0.44–1.54	0.54

*Model 6*			
Age	1.02	0.99–1.04	0.17
Height	1.02	0.99–1.05	0.19
SOFA score	1.03	0.94–1.12	0.57
Catheter position (jugular vein)	0.59	0.28–1.26	0.18
CVVHDF	1.13	0.51–2.52	0.76

ACT, activating coagulation time; Alb, albumin; CI, confidence interval; CVVHDF, continuous venovenous hemodiafiltration; Hb, hemoglobin; HD, hemodialysis; HR, hazard ratio; SOFA score, sequential organ failure assessment score.

## Data Availability

Data from this study are available from the corresponding author on request.
